# Editorial: Advances in cardiovascular medical technology

**DOI:** 10.3389/fmedt.2023.1309784

**Published:** 2023-10-30

**Authors:** Kevin Willy, Philipp Maximilian Doldi

**Affiliations:** ^1^ Department of Cardiology, University Hospital Münster, Münster, Germany; ^2^ Medizinische Klinik und Poliklinik I, Ludwig-Maximilians University, Munich, Germany; ^3^ Heart Alliance, Partner Site German Center for Cardiovascular Disease (DZHK), Munich, Germany

**Keywords:** wearables, medical technologies, interventional cardiology, artificial intelligence, machine learning

**Editorial on the Research Topic** Advances in cardiovascular medical technology

## Keeping fingers on the pulse of time—the ongoing technological transformation of cardiology

Constant technological progress and its implementation into clinical practice is vital for optimal medical care. The discipline of cardiology has always been characterized by its inventive solutions to clinical questions and the continuing optimization of procedures by new technologies. To account for these current developments, this Research Topic was dedicated to cardiovascular medical technology. We received a variety of manuscripts addressing different aspects of technological progress in medicine. Topics ranged from conservative medicine with the establishment of a single use stethoscope to avoid pathogen transmission (Nazari-Shafti et al.) to basic science projects analyzing the migration of endothelial cells on the surface of implantable vascular stents (Wang et al.) or the influence of stent morphology on blood flow (Kohata et al.). Further projects aimed at summarizing recent advancements in cardiovascular imaging (Counseller and Aboelkassem), stress quantification by wearables (Velmovitsky et al.) or smartphone gyrocardiography for heart rate monitoring (Elgendi et al.).

Especially in the era of digitalization, cardiology is one of the most affected disciplines in human medicine. One of the most popular novelties might be the use of wearables and smartphones to track physiological data such as heart rate, which can be assessed by smartphone gyrocardiography (Elgendi et al.), blood oxygen saturation or other parameters such as ECG or blood pressure. Especially for the detection of arrhythmias such as atrial fibrillation, wearables have already shown to deliver excellent results in finding the underlying cause for clinical symptoms such as palpitations or tachycardia (1, 2). Even more dangerous arrhythmias such as ventricular tachycardia or higher degree AV blocks have been reported to be reliably detectable with wearables (3). Future directions of research do not only aim at identifying arrhythmias but also to use detected physiological data as surrogates for physical or mental stress (Velmovitsky et al.) or clinical events such as heart failure decompensation. A recent JACC scientific statement underlined the crucial role of remote monitoring using several devices (implanted devices such as defibrillators, health care apps for the monitoring of vital parameters such as body weight/blood pressure/heart rate and options for telemetric counseling of heart failure nurses and cardiologists) to guide optimal therapy (4). The derived data are not only of clinical but also of scientific interest. Machine-Learning and other artificial intelligence approaches are frequently used in cardiology, focusing especially on ECG interpretation and the correct detection of arrhythmias to guide medication such as oral anticoagulation or the intake of antiarrhythmic drugs (5). Especially large artificial neural networks trained with thousands of datasets are of particular interest but the diagnostic criteria are often not obvious for users and the clinical benefit for the application remains to be proven.

A very essential point of digitalization in cardiology is that patients can be identified early as promising candidates for certain technological monitoring or modern interventional therapies. Many fields of cardiology have successfully applied digital interventions to increase the effect of cardiac rehabilitation (6) or heart failure management (7) using psychological interventions and digitally guided exercise training. Especially in times of understaffing in the health sector, these interventions have the potential to keep up and even strengthen health care provision especially in rural areas and less privileged countries.

The rapidly increasing progress in science and technology has not only enriched our daily practice with wearables and artificial intelligence, but also highly revolutionized the section of cardiovascular imaging. Especially in the prospering field of valvular heart disease, a timely and accurate diagnosis is crucial for effective management and treatment of these conditions. Over the years, significant progress has been made in cardiovascular imaging techniques, such as 2D and 3D echocardiography, cardiac MRI, and more. Most recently the accuracy and prognostic relevance of rather novel technique such as 4D MRI flow imaging or echocardiographic strain imaging have been demonstrated (8, 9). These advancements have revolutionized the field of cardiology by providing clinicians with a wealth of detailed information about cardiac structure and function (Counseller and Aboelkassem). Especially in the context of valvular heart disease such as mitral- and tricuspid regurgitation, the progress in medical technology and cardiovascular imaging is striking. In particular, in the growing elderly population, these diseases have caused a significant burden of symptoms leading to lower physical capacity, frequent hospitalizations, deprivation of quality of life and mortality. The advances in medical technology within the last years have allowed for the exponential growth of treatment options for this large high-risk patient population (10, 11). Within the last couple of years, we have not only been provided with new and effective treatment tools, but also with a broad variety of outcome predicting diagnostic elements (12–15). These advances not only guide physicians in their decision making, but also help to early identify patients at risk.

One crucial aspect about the advances in medical technology has to be emphasized in particular: The immense potential of these new technologies in the context of preventive medicine (16, 17).

In conclusion, the dynamic intersection of technology and cardiology has yielded remarkable advancements that are redefining the landscape of cardiovascular care. From the adoption of wearables and smartphones for precise physiological data tracking, including heart rate and arrhythmia detection, to the implementation of artificial intelligence in ECG interpretation, the digital era has ushered in a new time period of patient engagement and clinical decision support. The impact of digital interventions, particularly in cardiac rehabilitation and heart failure management, extends the reach of healthcare, bridging gaps in access and promoting patient involvement.

Moreover, the remarkable progress in cardiovascular imaging, including 2D and 3D echocardiography and cardiac MRI, has armed clinicians with insights into cardiac structure and function. This revolution is especially significant in valvular heart disease, offering timely diagnosis and tailored treatment options, ultimately improving the quality of life and prognosis for patients.

While these technological innovations have brought us tools for diagnosis and treatment, they also hold great promise in the realm of preventive medicine. The potential to identify at-risk individuals early on signifies a transformative shift towards proactive healthcare. As we journey forward, embracing these advancements will undoubtedly empower both clinicians and patients, promising a brighter and healthier future for cardiovascular medicine.
